# Hospital and Institutional News

**Published:** 1915-06-05

**Authors:** 


### HOSPITAL AND INSTITUTIONAL NEWS.

### NATIONAL SERVICE INDEED.

We direct the attention of our readers to two articles of importance in the present issue, that on "National Service and the Hospitals," and that on "The Shortage in Hospital Medical Staffs." If the practical suggestions contained in these articles find general acceptance by the War Office and the voluntary hospitals, the improvement and saving which must result may prove momentous. The Special Hospital Sunday Number of The Hospital, which is issued this week, and will be largely distributed amongst the churches and places of worship throughout London, contains two fine illustrations of splendid work amongst the wounded which the voluntary hospitals are doing. One full-page illustration proves the ready adaptability to the circumstances of the war which hospital managers throughout the country have shown. It is a striking and most appealing picture, and we hope to give our readers an opportunity of studying it in The Hospital itself next week. What admission to a voluntary hospital means to the wounded officers and men was strikingly shown in a visit the Editor paid to a great hospital this week. He found a number of officers in bed on one of the balconies. One of these officers ended a brief conversation with the heartfelt utterance, "I am fortunate to find myself here." Let these few words convey to all the zealous workers throughout our hospitals the feeling of gratitude which their unselfish and untiring services have everywhere created in the hearts of sailor and soldier patients of all ranks in both Services.

### OUR "HOSPITAL SUNDAY" APPEAL NUMBER.

We publish this week, in accordance with customary practice, our annual Hospital Sunday Appeal Number. It appears as usual ten days in advance of Hospital Sunday itself, which falls this year on June 13, and is distributed to all the churches of the Metropolis in time for the congregations to master the special needs of the hospitals described in it before the actual appeal in the churches is made. In view of the momentous circumstances in which that appeal is made this year, and of the special work of the voluntary hospitals in connection with the wounded, opportunity has been taken to present and summarise that work in a vivid and striking manner. The Appeal Number, therefore, opens with a first-hand account of the war work in the London hospitals from the time of the first arrival of wounded to the end of April, and to give a vivid picture of this as a history and in living detail, St. Thomas's Hospital is taken as a typical example and made the centre round which the story unfolds. Illustrations are given of the more striking aspects of the work, and then follows a careful summary of the needs of the London hospitals in the light of the facts outlined in the opening article. Any reader can thus see at a glance where any hospital he (or she) particularly favours needs help at the present time. An article, entitled the "Hospitals' Annual Budget," deals with finance, and a series of telling diagrams in an article headed "The Roll-Call of the Sick" show at a glance how the hospitals deal with every type of case. That Hospital Sunday is also the festival on which the Gospel of Personal Service is annually preached is not forgotten. A special article, entitled "The Gospel of Personal Service," deals with the lesson of Hospital Sunday in this sense, and concludes with a word to the clergy, one of whom founded the movement, reminding them that Hospital Sunday stands for Christianity and Churchmanship at work in the world. Copies of the Appeal Number, price one penny, can be obtained on application to The Manager, The Scientific Press, Limited, 28 and 29 Southampton Street, Strand.

### ANOTHER SECRETARY KILLED IN ACTION.

Another hospital secretary has fallen in action, and we have to record with deep regret the death of Captain J. W. Garnier. He first entered hospital life as steward at the *Dreadnought* Hospital, Greenwich, in 1907, and after having served two and a half years he was appointed secretary to the Hastings, St. Leonards, and East Sussex Hospital. Here he did excellent work, and his energy and skill went far towards promoting the scheme for the reorganisation and rebuilding of the institution. On the outbreak of war Captain Garnier was called up for service, and a short time ago was grievously injured. He died of wounds on May 28 at St. Gabriel's College Hospital, Camberwell, at the age of thirty-seven. Captain Garnier was an officer of the 3rd Royal West Surrey Regiment (The Queen's). He served in the South African War, taking part in the operations in Cape Colony, and received the Queen's medal with three clasps. He was the second son of the late Rev. T. P. Garnier, Hon. Canon of Norwich, and of the late Hon. Mrs. Garnier, daughter of the fifth Lord Vernon.

### PRECAUTIONS AGAINST POISONOUS BOMBS.

The possibility that in a future air raid on London bombs containing poisonous gases might be exploded has already been hinted at by the police authorities, and many people are no doubt asking their doctors what specific precautions it would be advisable to take in such an event. Very much, of course, depends on the nature of the gas employed; but, if any true idea can be obtained from the experiences of the men at the Front, there is clearly reason to expect the use of bombs containing liquefied chlorine or bromine. As a protection against fumes from either of these, respirators of the cotton-wool type treated with solutions of common washing soda or of hyposulphite of soda—generally known to amateur photographers as "hypo"—are no doubt the best. Strong solutions should be used, the respirator being soaked in them and the excess of liquid squeezed out. The reason for using these salts is, of course, that they combine with chlorine to produce harmless compounds. A "portable" respirator could be made by saturating the wool with a strong solution and allowing the respirator to dry; the dried respirator could then be carried in the pocket and moistened with ordinary water before use. It is, however, quite easy to exaggerate the danger from the liberation of chlorine in London, where the conditions are in no thinkable way in the slightest degree similar to the conditions in the trenches; when the gases were first used in the fighting line the men were forced either to expose themselves to fire or to suffer the effects of the poisonous fumes. It would seem that more harm might result from anticipation of the danger from poison-bombs exploded in London than could actually result from the fumes.

### TWO BEDS ENDOWED AT KING EDWARD VII.'S HOSPITAL, CARDIFF.

ALDERMAN W. H. MATHIAS, J.P., of Tyn-y-cymmer Hall, Porth, recently visited King Edward VII.'s Hospital, Cardiff, accompanied by his nephew, Sir William James Thomas, to see the patients, including the wounded soldiers. He was so much impressed by all he saw at the hospital that before leaving he handed to the secretary a cheque for 2,000 guineas to endow in perpetuity two beds in the "Coronation" Ward—one in memory of his wife, the late Mrs. Rachael Mathias, who died a few months ago, and the other in memory of his son, the late Dr. Richard Mathias, M.A., M.B., of Radyr, who died some three years ago. Dr. Mathias was, like his father, a life president of the hospital. Alderman Mathias wishes his gift to date from May 25, as that would have been his golden wedding day. To no person will the gift give greater pleasure than to Sir William James Thomas, as the late Mrs. Mathias was his aunt, being the eldest daughter of the late Mr. James Thomas, of Ynyshir. It may be stated that Alderman Mathias has placed his late residence, Clydach Court, Pontypridd, at the disposal of the committee of No. 9 Ward, Ynyshir, and in this house, with its beautiful gardens, thirty-three Belgian refugees are now accommodated.

### THE CHAIRMAN OF THE BELGIAN FIELD HOSPITAL RESIGNS.

MR. HAROLD HODGE, formerly editor of the *Saturday Review*, has resigned the chairmanship of the Belgian Field Hospital. His explanation for taking this course of action is given in a published letter from which we take the following extracts. In an appeal issued in January the institution was described as "the only British hospital acting under the directions of the Belgian War Department." "But, quite recently," writes Mr. Hodge, "that department has required that the head of the hospital's medical staff shall be a Belgian, responsible to the Belgian authority, not to the committee of the hospital, thus ousting the distinguished English surgeon-in-chief appointed by the committee. In these circumstances the hospital ceases to be British, the medical staff and its control being of the *esse* of a hospital. Therefore it seems to me not honest to use money given to a British hospital for one that is no longer British—at any rate, not British in at all the same sense—and still less can it be right to go on receiving and asking for more money without informing subscribers and the public of the change in the hospital's constitution. Further, by this change the committee become responsible for an officer whom they cannot control. Also, the present commandant being a layman and unversed in things medical, the committee, minus a chief surgeon responsible to them, can have no regular means of getting information as to the medical conduct of the hospital. This seems to me a position morally false and administratively indefensible for any hospital committee to accept." Mr. Hodge concludes his letter with the following suggestive paragraph: "My natural regret at parting from a piece of war work with a rather remarkable record is, I admit, qualified by serious doubts whether this hospital has not now fulfilled its proper mission. I think it possible that it has done its work and that the resources and energy bestowed upon it might perhaps be turned to better account otherwise."

### TRIBUTE TO OUR STRETCHER-BEARERS IN THE FIELD.

THE veil which has so far hung over the individual exploits of all ranks except of those few who have actually been rewarded by the V.C., or other distinction, is gradually being lifted, and this change has come about by the slightly increased opportunity allowed to newspaper correspondents at the Front. Thus we find in Mr. John Buchan's article in the *Times* which described the operation at Festubert a whole paragraph devoted to the examples of bravery exhibited by stretcher-bearers on that occasion—names and all. "Brilliant was the work done by our stretcher-bearers," he writes. "Private Williamson, of the Queen's, under machine-gun and shell fire, carried back wounded, and when it seemed impossible for him to return he refused to take cover, and went back to the firing-line. Similar feats were performed by Corporal Tilson, of the Warwicks, and by Corporal Coleman, of the Border Regiment. The latter, although the colonel's servant and exempt from this duty, brought back no fewer than fifty men." Such accounts cannot, and do not, pretend to be complete, but they make the relatives at home, and the village, factory, office, or college with which the hero was associated, proud and emulative, and bring home the war in a small way which helps to mitigate the effect of long casualty lists. Hospital workers and Red Cross men at home will be glad to know that their comrades are as good in the firing-line as in the hospital.

### LONDON AND THE BELGIAN RED CROSS.

FROM June to October a War Exhibition is being held at Prince's Skating Club, Knightsbridge, S.W., on behalf of the Belgian Red Cross, by means of which London will have a great opportunity of understanding—almost as by a visit to that country—our obligation to the Belgian nation; while at the same time the extent to which science and industry are being employed in the enormous struggle is illustrated by the exhibits, some of which make a direct appeal to hospital and institutional workers. There are seven sections in the War Exhibition, five of which may be briefly noted, but the remaining two are of technical hospital interest. "Trophies of War" from important battle-grounds; "Armament and Munitions in the Making"; "Science and Industry," as applied to war; "Equipment," in which, by the way, equipment for hospitals and field service is included; and a "Maritime and Aerial" section, comprising the latest inventions in naval and aerial warfare, provide the subjects for five sections. Section No. 3, however, is devoted to "Red Cross Work, Hospital and Nursing," in which the vital part played by women in the war is elaborately illustrated. There is also a "Food and Hygiene" section (No. 5), devoted to the display of the specialties of all firms who supply foodstuffs to the War Office, including foodstuffs and special comforts for wounded and invalided men, and convalescents. Part of this section will illustrate the hygienic preparations and apparatus employed—a vast subject in itself. Apart from the customary exhibition orchestra and entertainments there is a mural panoramic representation of Belgium, covering nearly 14,000 square feet. Bruges, Louvain, the valley of the Meuse, and the old and new historic places, with the beauties of Belgium, are displayed upon it. Season tickets are obtainable at one guinea each, and communications should be sent to the Organising Secretary, War Exhibition, London Chamber of Commerce, 97 Cannon Street, E.C.

### HOSPITAL WORK AND PARLIAMENT.

The Cabinet changes which have resulted in the so-called Coalition Government present one or two points of interest to the hospital world which are worth a passing attention. Mr. Samuel gives up his post as President of the Local Government Board and returns to the Post Office, a portfolio now placed outside the Cabinet, although mention has been made of his still being associated with work on behalf of the Belgian refugees. The new President of the Local Government Board is Mr. Walter Long, who, under present circumstances, is not more likely than anyone else to disturb such noiseless tenor in his department as the problems affecting it owing to the war may permit. While there is no particular importance in these facts to hospital managers, such members of the British Medical Association as are not too busy on war work to spare a glance at this revision of the political spoils will note with interest, together with their colleagues on the non-panel and panel societies, that room has apparently been found for Dr. Addison in a new post. He is therefore leaving his position at the Education Office, and takes up the duties of Under-Secretary to the new Minister of Munitions, who, it will be recalled by medical men, is his old friend Mr. Lloyd George. Dr. Addison's "push and go," crowned, as we all remember, by a dinner in his honour at which Mr. George presided, is therefore receiving more important and more practical recognition. There should be plenty of scope for energy and driving, not to say cajoling power, in his new post, and it is, no doubt, for the proofs which he has given of these qualities that Dr. Addison has been appointed to it.

### PROMOTION IN MENTAL HOSPITALS.

Some local surprise has been caused by the unexpected resignation of the medical officer of Fife and Kinross District Mental Hospital at Springfield, Dr. J. J. Harrower-Ferguson. The appointment, which is in the hands of the Fife and Kinross District Board of Control, was filled by Dr. Harrower-Ferguson only last November. Educated at London University College, he graduated at Edinburgh University in 1906. A year later he became junior medical assistant at Springfield, and shortly afterwards senior assistant. On Dr. Turnbull's retirement last November, he was appointed to his present post, which carried a salary and emoluments of the value of £600 a year. As the retiring medical officer is only thirty-three years of age, his advancement in the mental hospital service must be regarded as unusually rapid, and though, naturally, chance can play a large part in matters of this kind, such rapid promotion is rarely possible, except in cases like the present, in which there is no rule or custom to preclude the senior administrative post from being given to the junior officer. The attitude which, in many hospitals, regards a junior officer as ineligible for promotion to the principal post, unless and until he has held a position of equal status to it in another institution, and, therefore, has additional experience, has much to recommend it. The virtue of new brooms, the possession of varied experience in the chief officers, are unmistakably advantageous to institutional well-being. But, if we mistake not, the rewards of the mental hospital service have been so few and far between, the monotony of mental hospital life has been so generally admitted, that men in the junior posts have secured, as it were, more of a prescriptive right to promotion in their own institutions than has been the case in other branches of hospital work.

### NEW SUPERINTENDENT OF FIFE MENTAL HOSPITAL.

Dr. Ferguson's successor as medical superintendent of the Fife and Kinross District Mental Hospital at Springfield was announced last week after a special private meeting of the District Board of Control. The Board has appointed Dr. James H. Skeen, medical superintendent of Kirklands Mental Hospital, to fill Dr. Ferguson's post. The salary has been fixed at £600 per annum, which is an increase of £120 on that paid to the late superintendent; and a house and other emoluments are valued at £120 a year. Educated at Aberdeen University, Dr. Skeen became assistant medical officer at Stirling District Mental Hospital. He has held his present post at Kirklands for twenty-one years.

### SUICIDE IN A NAVAL HOSPITAL.

The only case of suicide in twenty-five years was revealed at an inquest held last week at the Royal Naval Hospital, Yarmouth, into the death of a patient, aged forty-nine, who had been an able seaman of the Royal Fleet Reserve, and was discovered strangled by his braces. Some interesting details of the administration of the hospital were given in the course of his evidence by Fleet-Surgeon Miller, medical officer in charge. He produced a tell-tale recording apparatus kept in his office, which "explained" that on a chart actuated by a clock each attendant indicated that he was performing his duties every hour. Each attendant, moreover, had signed a book indicating that he knew that the deceased was on the suicidal list of patients. The official number of beds at the hospital is 227, and it was stated that there are thirty or thirty-one attendants at the hospital for 150 patients, being an average of one to every five. Despite the vigilance implied by this system, the deceased had managed to leave his ward and pass along a corridor leading by way of a lavatory to a stair-head, where he fastened his braces to an iron-netting support on the stair-rail. He had been admitted on March 19 as a certified lunatic; and, as a result of being seen with a towel round his neck on May 5, was placed on the suicidal list. The tell-tale showed that the attendant was awake and watchful, for the record proved that a signal was sent twice each hour from the deceased's ward, in which three attendants were available in case of need. The evidence of attendants showed that the deceased had exercised much cunning, having left his pillow in his bed and arranged his clothes so as to suggest he was still there, and presumably crawled to the door along the floor, having smuggled his braces under his shirt when the patients' clothes were removed for the night. He suffered from the delusion that he was going to be shot for some imaginary crime. The Coroner said there was no doubt that the deceased had committed suicide while insane, and the jury considered that no blame attached to anyone.

### A DILATORY BOARD AND THEIR MEDICAL OFFICER.

The Barnet Board of Guardians have again had a gentle reminder that their favourite policy of postponement does not always commend itself to the officers, even if it has their approval. Several children in their scattered homes have been affected with measles, and have been removed to the workhouse, a most unsuitable place. Two children had died, and the board still decided to let the whole affair slide. However, the medical officer wrote: "I cannot state too strongly to the board the serious light in which I view the admission of infectious cases to the workhouse, which is without an isolation building or adequate accommodation, and which is already overcrowded. I would urge the board to define the position clearly, in order that there may be no misunderstanding on the subject in the future. As an alternative, the Guardians may possibly prefer me to ask the Local Government Board for their ruling on the matter." This letter at least had the effect of forcing the board to decide that the disused nursery at the workhouse should be used as an isolation ward. It is a matter for grave concern that Boards of Guardians so close to London, as is the case with Barnet, should still have antiquated conceptions of what is their duty to the sick poor, whose only protection under such circumstances are letters similar to that of the medical officer quoted above.

### PATIENTS' PAYMENTS AT BRIDGWATER HOSPITAL.

A special meeting of the subscribers to Bridgwater Hospital was held last week to consider certain recommendations put forward by the committee of management, the chief of which proposed to revert to the system of charging for patients. The meeting was called in view of the serious financial position of the hospital, which may be judged from the facts adduced by Mr. H. W. Pollard, chairman of the committee of management. In moving, therefore, that in future all patients over fourteen years of age should make a payment of 3s. per week as in-patients and 1s. per week as out-patients, while those under fourteen years of age should be charged half these amounts, he pointed out that accident and casualty patients would still be admitted free. In his speech Mr. Pollard remarked that the extension now being carried out had cost a total of about £6,000, of which £4,000 had been taken from the reserve funds. The annual income of the hospital was thus reduced by £160. He then pointed out that the workmen's subscriptions had fallen from £275 a year five years ago to £42 last year. Meanwhile the running expenses had been increased by the rise in the cost of provisions and drugs. To improve the income was, therefore, a matter of urgency and one direction of doing so was charging for patients. The proposal was carried, and a committee of local ladies was also formed to support the working of the hospital, and it was suggested that it might also canvass for subscriptions.

### THE ROOT OF THE FAILURE AND THE WAY OUT.

Since the above decision to charge patients has been come to, we may commend to Mr. H. W. Pollard and his committee a detailed article describing how patients' contributions were introduced at King Edward VII.'s Hospital, Cardiff, contributed by the Secretary, Mr. L. D. Rea, which appeared in The Hospital of December 30, 1911. The process of introduction and the machinery for collecting the payments of both in- and out-patients is lucidly described, and anyone studying the details may learn not merely a useful system, but how to introduce it without friction by gaining the support of the patients themselves. We confess, however, that a more pregnant suggestion seems to us to lurk in the remarks of Mr. C. E. Pierson, of Burnham, who presided. While seconding the resolution, he pointed out that some method should be adopted for bringing the advantages of the hospital before the public which it serves. That this has not been properly done seems indisputable from the disastrous fall in the workmen's contributions, which are now, on Mr. Pollard's showing, all but non-existent. This fact cannot be regarded otherwise than as a reflection on the organisation of the collections; and this matter should be taken in hand at once, for all experience proves that no patients' payments or other special means can make good any deficiency in local interest and support, to consolidate which by hard and steady work is, and must always remain, the basis of voluntary hospital finance. On such work alone can any hospital build up a secure financial position.

### MEDICAL DEFENCE UNION AND LEICESTER INSURANCE COMMITTEE.

At a meeting of the Leicester Borough Insurance Committee held last week, presided over by Mr. W. Higginson, the Finance and General Purposes Sub-Committee reported that a writ had been served on the Insurance Committee by the Medical Defence Union on behalf of Dr. C. W. Moore, who was on the Committee's panel up to December 31. The main object of the writ was reported to be to restrain the Committee from surcharging the doctor with the sum of £31 7s. under certain sections of the medical benefit regulations. Mr. W. S. Rosie, who moved the adoption of the report, stated that the Insurance Commissioners advised the Committee to defend the action, and promised them their moral support. Another speaker declared that it would be a serious matter if the action went against the Committee, because the medical men would be able to "kill them" with the cost of extravagant prescriptions. The Chairman said that it was possible that the High Court would refer the case for arbitration. He thought the case would not have gone so far if the writ had not the sympathy of the local medical men. To this remark, which was made in answer to a question, another speaker replied that the local medical committee knew nothing about the writ. The report was then adopted.

### A LONDON PARK FOR HOSPITAL PURPOSES.

The first park under the control of the London County Council to be closed to the general public is Myatt's Fields, Camberwell, since the ground is required by the War Office for temporary hospital buildings. The park, or fields, run to about fourteen acres, which were laid out as a park about twenty-five years ago, when £10,000 was spent upon them. Various portions of the other public parks of London have been devoted to military purposes and hence closed to the public, but Myatt's Fields was entirely appropriated for temporary hospital buildings on Monday last. We shall not be surprised if the admitted success of the work which is being accomplished in the best designed temporary hospital structures does not give an impetus to hospital architecture after the war in respect of inexpensive buildings. The change is one which would have much to commend it, since expensive hospital architecture has largely sprung from the desire of the medical profession to enhance the dignity of their medical schools.

### BOARDS OF GUARDIANS AND SOLDIERS IN HOSPITAL.

At a recent meeting of the Huddersfield Board of Guardians the question came up as to the amount of the subscription which the Board should give to the Royal Infirmary. A resolution was moved and seconded to the effect that the Board should pay to the Huddersfield Royal Infirmary the sum of £100. Alderman Wheatley then moved that the amount should be £250, on the ground that the infirmary greatly needed increased support because of the presence in hospital of wounded soldiers. This was seconded, and it was also pointed out that the amount would be subject to the approval of the Local Government Board. After discussion, Alderman Wheatley withdrew his amendment, but gave notice that at the next meeting of the Board he would move that a grant of £150 be made to the infirmary. The resolution was therefore agreed to. At a joint meeting, held later, of the committees of the Crosland Moor and Deanhouse institutions it was reported that the Board had been asked to accommodate sixty wounded soldiers at the former, and that arrangements had been made for doing so by transferring the whole of the patients in No. 1 hospital block to Deanhouse Hospital. The joint committee approved of this action and appointed representatives of the two committees as a committee to take any further necessary steps. It would thus appear that the Huddersfield Guardians are fully alive to the necessary work of attending to the wounded, in which, in common with the infirmary, they are doing their share. It is therefore to be hoped that the Guardians may see their way to increase their subscription to the infirmary, which, in the interests of the soldiers, the Guardians, and, not least, its ordinary civil patients, deserves all the support that can be given to it.

### THE SCIENTIFIC ORGANISATION OF MILITARY MEDICINE.

The Medical Research Committee in relation to National Health Insurance, which was established a year before the outbreak of war and then placed its services at the disposal of the War Office, has issued a very interesting interim Report on its work in relation to military medicine. The White Paper embodying this Report has been laid before Parliament, and the different branches of the Committee's work form very instructive reading. The Committee, it will be remembered, had framed last year an extensive scheme for research in tuberculosis and other diseases; but its war work is what now claims attention. The treatment of infected wounds, as we all know, has been investigated by Sir Almroth Wright at Boulogne, and a memorandum based on his researches has been addressed by the War Office to all medical officers with the Forces, thus realising, to some extent, Sir Almroth's wish for centralised instructions in relation to the treatment of wounded, which was described in our columns in the course of a report of his recent lecture on the septic infection of wounds. In short, the whole of the Medical Research Committee's bacteriological department has been applied to problems of military importance. The Committee's statistical department is also, at the disposal of the War Office, engaged in compiling statistics of the sick and wounded which will be of enormous value after the war in regard to the health of the male population, apart from their importance to military medicine. The Committee, with the special fund luckily set aside for research by the Government before the war, has sent an expedition to Galicia to secure cultures of cholera vaccines; it has provided workers for the study of cerebro-spinal meningitis; it is arranging for the better co-ordination of the studies of trained observers of important classes of cases in the military hospitals. This is but an indication of the vital interest of the work, to which we hope to return in greater detail.

### CHARITY FETES IN WAR-TIME.

Apart from Hospital Sunday, few charity fêtes are surviving the public absorption in the war, but an exception, of course, has to be made in respect of fêtes and exhibitions designed to benefit the British Red Cross Society. Thus the success of the Christie's sale of art works in April on behalf of the fund in aid of the wounded (which realised £38,215), is probably inspiring those who are organising an exhibition of antique silver in aid of the British Red Cross Society and the Order of St. John. The exhibition, to be held from June 7 to June 18 at Messrs. Garrod's showrooms in Albemarle Street, will include examples from the principal collections in the country, and the Royal Family will be among the contributors. There is also to be a fête on behalf of the same joint fund at Ranelagh early in July. The fête has this unique fact about it that it will be the first occasion on which the committee of the club has lent its grounds for the benefit of a charity, and the first time that a charitable function has been held there. This is a striking tribute to the general enthusiasm which Red Cross work is exciting, and to the allied fact that the only charities which have much chance of maintaining their customary income from social festivities are war charities, which inevitably prove to be those associated with hospital work. This fact should, we think, encourage hospital managers not to indulge in cheap pessimism, but to realise that for institutional committees who know how to handle the situation, hospital work is receiving a deserved and gigantic advertisement by means of which any well-advised appeal is likely not to prove a failure. Even if such an appeal is not primarily concerned with work for the troops, their presence in the wards is having so powerful an influence on hospital work of all sorts that appeals even for building funds and old debts can be legitimately associated with the cause, which is so rightly dear to the public.

### DEATHS UNDER ANÆSTHETICS.

Embedded in that bulky Blue-book which contains the Annual Report of the Registrar-General of Births, Deaths, and Marriages are some interesting statistics relating to deaths by poisoning, and an examination of these and a comparison with the statistics for previous years show that there is little variation in the mortality rate from this cause from year to year. Apart from deaths under anæsthetics, the number of fatalities due to poisonous substances is between seven and eight hundred annually. During 1913, the year with which the recently issued Report deals, there were 190 fatal cases in which poisonous substances were taken accidentally, while in 470 cases the poisons were taken by suicides. Among the "accidental" cases the poison which accounted for the greatest number of deaths was laudanum, no fewer than forty-eight deaths being caused by this poison or by some other preparation or derivative of opium; to carbolic acid taken in mistake for some harmless substance ten deaths were due, and to preparations of belladonna the same number. In the category of suicides hydrochloric acid heads the list with a death-roll of 98; oxalic acid comes next with 88, and carbolic acid next with 64. The number of deaths under or connected with the use of anæsthetics was 296, against 283 in the previous year. In 111 cases the kind of anæsthetic is not stated, but in the remaining cases the anæsthetics are specified as follows: chloroform, 110; chloroform and ether, 25; ether, 25; A.C.E. mixture, 7; nitrous oxide, 7; ethyl chloride, 5; hedonal, 2; cocaine, 2; alcohol and chloroform, 2; stovaine, 1. The above statistics are of much the same character as in previous years, and nothing calls for special comment.

### THIS WEEK'S DRUG MARKET.

Business has now recovered from the effects of the Whitsuntide holidays, and the drug market is again fairly active. Prices are, with very few exceptions, well maintained. There is a good demand for bromides, and another advance in price is not improbable. The value of opium is well maintained, and makers of morphine and codeine continue to be very busy. Strychnine is again dearer in consequence of the scarcity of nux vomica. Cascara sagrada has an upward tendency in price. Quinine continues to be in good demand, and prices tend upwards. One of the few drugs which have an easier price tendency is acetanilide, the reason being that the drug is offered more freely. Otherwise the values of synthetic drugs are firmly maintained, and salicylates are again rather dearer. Business has been done in camphor, and prices tend upwards. Quicksilver has advanced in price, and quotations for corrosive sublimate, calomel, and other salts of mercury may be re-arranged accordingly. The market for Italian drugs is being watched with interest, but so far there have been no very remarkable price movements in such drugs, the advances in the prices for citric acid, cream of tartar, and tartaric acid not having been very pronounced up to the present; but higher prices for these articles may be looked for. Essence of lemon is still quoted at much the same prices, but any change would no doubt be in an upward direction. The prohibition of the export of olive oil from Italy can hardly fail to influence its price.

